# Tailoring PTV expansion to improve the dosimetry of post modified radical mastectomy intensity‐modulated radiotherapy for left‐sided breast cancer patients by using 4D CT combined with cone beam CT

**DOI:** 10.1002/acm2.13244

**Published:** 2021-05-02

**Authors:** Jiyong Zhang, Lei Huang, Fangcai Wu, Guoxi Wang, Lili Wu, Baotian Huang, Yan Lin, Dongsheng Li, Changchun Ma

**Affiliations:** ^1^ Department of Radiation Oncology Cancer Hospital of Shantou University Medical College Shantou China; ^2^ Department of Medical Imaging The Second Affiliated Hospital Shantou University Medical College Shantou China; ^3^ Guangdong Provincial Key Laboratory for Breast Cancer Diagnosis and Treatment Cancer Hospital of Shantou University Medical College Shantou China

**Keywords:** dosimetry, intra‐fractional CTV displacement, inter‐fractional CTV displacement, intensity‐modulated radiotherapy, left‐sided breast cancer, post modified radical mastectomy, tailoring PTV expansion

## Abstract

**Purpose:**

Our study aimed to improve the dosimetry of post modified radical mastectomy intensity‐modulated radiotherapy (PMRM‐IMRT) for left‐sided breast cancer patients by tailoring and minimizing PTV expansion three‐dimensionally utilizing 4D CT combined with on‐board cone beam CT (CBCT).

**Methods:**

We enrolled a total of 10 consecutive left‐sided breast cancer patients to undergo PMRM‐IMRT. We measured the intra‐fractional CTV displacement attributed to respiratory movement by defining 9 points on the left chest wall and quantifying their displacement by using the 4D CT, and measured the inter‐fractional CTV displacement resulting from the integrated effect of respiratory movement, thoracic deformation and set up errors by using CBCT. We created 3 different PMRM‐IMRT plans for each of the patients using PTV_t_ (tailored PTV expansion three‐dimensionally), PTV_0.5_ and PTV_0.7_ (isotropic 0.5‐ cm and isotropic 0.7‐ cm expanding margin of CTV), respectively. We performed paired samples t test to establish a hierarchy in terms of plan quality and dosimetric benefits. *P* < 0.05 was considered statistically significant.

**Results:**

The inter‐fractional CTV displacement (2.6 ± 2.2 mm vertically, 2.8 ± 2.3 mm longitudinally, and 1.7 ± 1.2 mm laterally) measured by CBCT was much larger than the intra‐fractional one (0.5 ± 0.5 mm vertically, 0.5 ± 1.0 mm longitudinally, and 0.3 ± 0.3 mm laterally, respectively) measured by 4D CT. Intensity‐modulated radiotherapy with tailored PTV expansion based on inter‐fractional CTV displacement had dosimetrical advantages over those with PTV_0.5_ or those with PTV_0.7_ owing to its perfect PTV dose coverage and better OARs sparing(especially of heart and left lung).

**Conclusion:**

The CTV displacement in PMRM‐IMRT predominantly arises from inter‐fraction rather than from intra‐fraction during natural respiration and differs in 3 coordinate axes either inter‐fractionally or intra‐fractionally. Tailoring and minimizing PTV expansion three‐dimensionally significantly improves the dosimetry of PMRM‐IMRT for left‐sided breast cancer patients.

**Abbreviations**
PTV: plan target volume
4D CT: four‐dimensional computed tomography
PMRM‐IMRT: post modified radical mastectomy intensity‐modulated radiotherapy
CT: cone beam CT
CTV: clinical target volume
OAR: organ at risk

## 1 INTRODUCTION

Our previous study had demonstrated that intensity‐modulated radiation therapy has dosimetrical advantages over three‐dimensional conformal radiotherapy with field‐in‐field technique (3DCRT‐FinF) and 2‐ partial arc volumetric modulated arc therapy (2P‐VMAT) for left‐sided breast cancer patients after modified radical mastectomy, and suggested that individually quantifying and minimizing CTV displacement might improve target dose coverage and heart and left lung sparing.^1^ Given the heart irradiation leading to the subsequent increasing risk of life‐threatening major cardiac events, including myocardial infarction, coronary revascularization, or death from ischemic cardiac disease,^2^, ^3^, ^4^, ^5^, ^6^, ^7^ and the nature of lack of a threshold value, their long‐term, dosage‐related effect as well as the additive nature of the risk with preexisting cardiac diseases,^8^ heart radiation exposure should be used as an a priori limitation parameter to evaluate which of the radiotherapy plans for left‐sided breast cancer after modified radical mastectomy is more advantageous if PTV dose coverage and other OARs sparing are acceptable.^1^ Our study aimed to verify the dosimetry improvement of post modified radical mastectomy intensity‐modulated radiotherapy (PMRM‐IMRT) for the patients by tailoring and minimizing PTV expansion three‐dimensionally utilizing four‐dimensional computed tomography (4D CT) simulation combined with on‐board cone beam CT (CBCT) verification under natural respiration.

## 2 MATERIALS AND METHODS

We enrolled a total of 10 left‐sided breast cancer patients undergoing IMRT after modified radical mastectomy in this study. Based on the contouring atlas published by the Radiation Therapy Oncology Group (RTOG),^9^ we delineated CTV including left chest wall and ipsilateral lymph node drainage area. A prescribed dose of 50 Gy/5w/25f was delivered to PTV. Each patient had 4D CT simulation before radiotherapy and three times of on‐board cone beam CT verification inter‐fractionally during the treatment. The intra‐fractional CTV displacement attributed to respiratory movement was measured by the 4D CT. The inter‐fractional CTV displacement resulting from the integrated effect of respiratory movement, thoracic disformation and setup errors was measured by the on‐board cone beam CT.

The study was performed in accordance with the Declaration of Helsinki, and was approved by the Ethics Committee of the Cancer Hospital of Shantou University Medical College. Informed consent form was obtained from each patient.

### 2.A 4D CT simulation

Each patient was placed in a supine position. Three lateral lines (labeled with 1, 2, and 3) were marked on the surface of the left chest wall using laser projecting across the lower edge of bilateral sternal heads, right nipple and the skinfold of the lower edge of the right breast, respectively. The other three longitudinal lines (named A, B, and C) were labeled across the left edge of the sternum, left mid‐clavicular line and left anterior axillary, respectively. The intersection points (A1, A2, A3, B1, B2, B3, C1, C2, and C3) of the three lateral and the three longitudinal lines were marked with ink and radiopaque metal beads on the surface of the left chest wall. The position changes of A1, A2, A3, B1, B2, B3, C1, C2, and C3 points measured by 4D CT were documented to reflect the intra‐fractional CTV displacement attributed to respiratory movement. For each patient, a helical CT scan was acquired under natural breathing followed by 4D CT scans taken at a representative normal respiratory cycle using a Philips Brilliance CT Big Bore Simulation System (Andover, MA). The respiratory signal was recorded with the Real‐Time Position Management (RPM) Respiratory Gating System (Varian Medical System, Palo Alto, CA) and synchronized with the CT data. The slice thickness was 3 mm at 512 × 512 pixels. Each image acquired and tagged to its corresponding phase of the respiratory cycle was then sent to the workstation using the 4D CT software. The position changes of A1, A2, A3, B1, B2, B3, C1, C2, and C3 points were documented to reflect the intra‐fractional CTV displacement attributed to respiratory movement. X, Y, and Z values indicated the displacement of the intersection points in x (laterally), in y (longitudinally) and in z coordinate axis (vertically), respectively.

### 2.B 3D on‐board cone beam CT verification

Each patient had a total of three times of on‐board cone beam CT verification inter‐fractionally randomly during the treatment. The CBCT scans were performed using the on‐board imager system installed on the Varian TrueBeam linac (Varian Medical System, Palo Alto, CA, USA). Under the half‐fan scan mode (field of view = 46 cm), the detector is centered laterally and longitudinally with respect to the source. The CBCT protocols used 1080 mAs at 125 kV with weighted CT dose index (CTDIw) of 1.60 Gy. The CBCT image acquired in half‐fan mode and reconstructed using manufacturer’s software. Patient position shifts in x, y, and z directions were documented after manually matching the radiopaque markers (the sternum and the labeled metal beads on the chest wall) between their initial simulation position and the present on‐board position.

### 2.C Plan comparison and statistical analysis

We created three different PMRM‐IMRT plans for each of the patients using the PTV_t_ (tailored PTV expansion three‐dimensionally), PTV_0.5_ and PTV_0.7_ (isotropic 0.5‐cm and isotropic 0.7‐cm expanding margin of CTV), respectively. All PMRM‐IMRT plans used two opposed tangential beams, and two anterior beams with a 10‐degree angle from the tangential ones, and a supraclavicular beam. The dose calculations were employed with a grid of 2.5 mm using the Anisotropic Analytical Algorithm (AAA). All plans were created with a prescribed dose of 50 Gy covering 95% of the PTV.

Clinical target volume (CTV) and OARs including heart, ipsilateral lung, spinal cord, and contralateral breast were contoured as previously^1^ using the Eclipse treatment planning system (Eclipse 10.0, Varian Medical Systems, Palo Alto, CA, USA). D_mean_, V_95%_, V_105%_, heterogeneity index (HI) and conformity index (CI) values were calculated for the PTV. V_95%_ was defined as the percentage of the PTV receiving 95% or more of the prescription dose. V_105%_ indicated the dose hotspot area that received 105% of the prescription dose. The heterogeneity index (HI) and conformity index (CI) were calculated as followed: HI = (D_2%_–D_98%_)/D_50%_, CI = (V_PTVref_/V_PTV_) × (VPTVref/V_ref_), where V_PTVref_ represents the volume of PTV covered with the reference dose. V_PTV_ represents the volume of PTV and V_ref_ represents the volume covered with the reference dose or higher. A lower HI value, ranging from 0 to 1, represents better homogeneity. A lower CI value, ranging from 0 to 1, represents worse conformity. D_mean_ is an average dose delivering to an organ. V_(xGy)_ represents the percentage of an organ’s volume receiving (x) Gy or higher. D_2%_ represented the dose corresponding to 2% PTV volume as shown in the dose volume histogram (DVH) and could be deemed as the maximum dose, whereas D_98%_ could be deemed as the minimum dose. D_50%_ represented the reference dose (or prescription dose) for PTV. D_mean_, V_5_ _Gy_, V_10_ _Gy_, and V_20_ _Gy_ were calculated for the heart and the left lung. We performed paired samples t test between any two of the three plans, to establish a hierarchy in terms of plan quality and dosimetric benefits. *P* < 0.05 were considered statistically significant. The SPSS 19.0 software (IBM, Chicago, IL) was used for statistical data management and analysis. *P* < 0.05 was considered statistically significant.

## 3 RESULTS

The intra‐fractional CTV displacement in PMRM‐IMRT for left‐sided breast cancer patients measured by 4D CT during natural respiration.

The overall intra‐fractional CTV displacement attributed to respiratory movement measured by 4D CT simulation differed in three directions, with 0.5 ± 0.5 mm vertically, 0.5 ± 1.0 mm longitudinally, and 0.3 ± 0.3 mm laterally, respectively. As exhibited in Table 1, the result indicated that the lower and lateral part of the chest wall tends to have a larger range of displacement than the upper and middle one during natural breathing. Either the displacement in y direction or in z direction was larger than in x direction during natural respiration (Table 1).

**Table 1:** The intra‐fractional CTV displacement in PMRM‐IMRT for left‐sided breast cancer measured by 4D CT.

Paramaters	X (mm)	Y (mm)	Z (mm)
A1	0.2 ± 0.2	0.4 ± 0.1	0.4 ± 0.4
A2	0.3 ± 0.3	0.5 ± 0.1	0.5 ± 0.4
A3	0.3 ± 0.3	0.4 ± 0.8	0.7 ± 0.5
B1	0.2 ± 0.4	0.5 ± 0.1	0.6 ± 0.6
B2	0.4 ± 0.4	0.5 ± 1.0	0.6 ± 0.6
B3	0.4 ± 0.4	0.5 ± 1.0	0.6 ± 0.5
C1	0.2 ± 0.3	0.6 ± 1.1	0.7 ± 0.7
C2	0.5 ± 0.4	0.5 ± 1.0	0.3 ± 0.3
C3	0.4 ± 0.3	0.5 ± 0.9	0.5 ± 0.4

The intersection points (A1, A2, A3, B1, B2, B3, C1, C2, and C3) of the three lateral and the three longitudinal lines were marked with ink and radiopaque metal beads on the surface of the left chest wall. The displacement of the intersection points was measured to quantify the position change of the left‐sided chest wall. X, Y, and Z values indicated the displacement of the intersection points in x (laterally), in y (longitudinally), and in z coordinate axis (vertically), respectively. Data presented as mean ± standard deviation(mm). Abbreviations: PMRM‐IMRT = post modified radical mastectomy intensity‐modulated radiotherapy. 4D CT = four‐dimensional computed tomography.

The inter‐fractional CTV displacement in PMRM‐IMRT for left‐sided breast cancer patients measured by CBCT during natural breathing.

The inter‐fractional CTV displacement in PMRM‐IMRT was much larger than the intra‐fractional one during natural breathing. The inter‐fractional CTV displacement resulting from the mixed effect of respiratory movement, thoracic disformation and setup errors, measured by on‐board cone beam CT were 2.6 ± 2.2 mm vertically, 2.8 ± 2.3 mm longitudinally, and 1.7 ± 1.2 mm laterally, respectively (Table 2). The CTV displacement predominantly arises from inter‐fraction rather than from intra‐fraction during natural breathing, and differs in three directions either inter‐fractionally or intra‐fractionally.

**Table 2:** Much larger inter‐fractional CTV displacement measured by CBCT than the intra‐fractional one in PMRM‐IMRT for left‐sided breast cancer during peaceful breathing.

Paramaters	Direction	Number	Mean ± SD(mm)	≤2 mm	≤3 mm	≤4 mm
Intra‐fraction	X	900	0.3 ± 0.3	99.7%	100.0%	100.0%
	Y	900	0.5 ± 1.0	88.1%	96.5%	100.0%
	Z	900	0.5 ± 0.5	96.5%	99.5%	100.0%
	Sum	2700	0.4 ± 0.7	94.8%	98.7%	100.0%
Inter‐fraction	X	30	1.7 ± 1.2	40.0%	93.3%	96.6%
	Y	30	2.8 ± 2.3	46.6%	60.0%	63.3%
	Z	30	2.6 ± 2.2	50.0%	66.6%	70.0%
	Sum	90	2.4 ± 2.1	45.5%	73.3%	76.6%

X, Y, and Z values indicated the inter‐ or intra‐fractional CTV displacement in x (laterally), in y (longitudinally) and in z coordinate axis (vertically), respectively. Data presented as mean ± standard deviation (Mean ± SD) (mm). The values in the last three columns were the CTV coverage rates under the corresponding status when using CTV expanding values with 2, 3, and 4 mm, respectively. Abbreviations: PMRM‐IMRT = post modified radical mastectomy intensity‐modulated radiotherapy. CBCT = cone beam computed tomography.

Dosimetrical advantage of PMRM‐IMRT using PTV_t_ over those using PTV_0.5_ or those using PTV_0.7_ for left‐sided breast cancer patients under natural respiration.

**Fig. 1:**
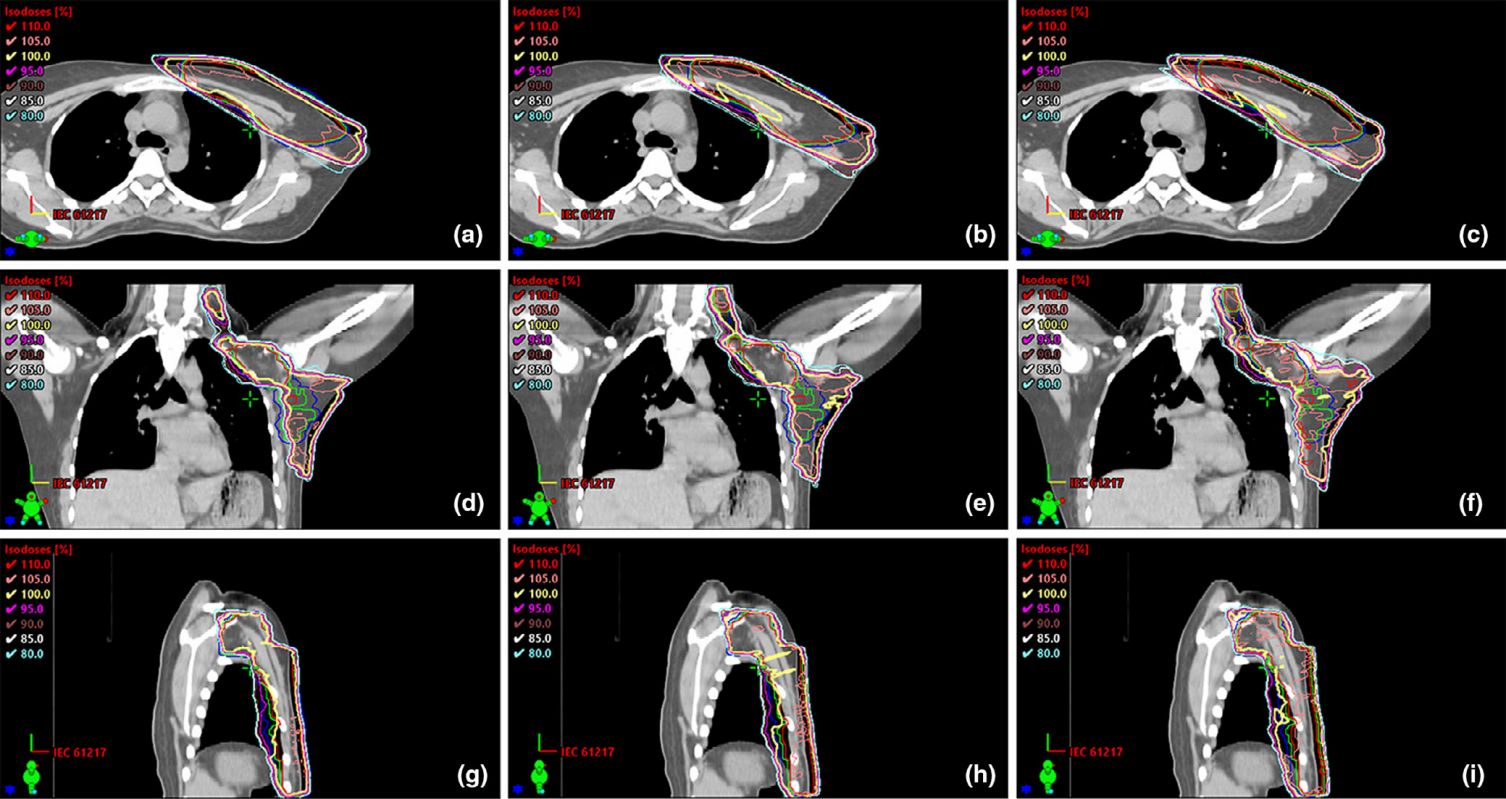
Transverse, coronal and sagittal dose distribution curves for the three PMRM‐IMRT plans using PTV_t_ (red), PTV_0.5_ (green), and PTV_0.7_ (blue) in a representative patient. (a, b, c), (d, e, f), and (g, h, i) exhibited dosage distribution in the transverse section, the coronal plane and the sagittal plane. PTVt (red), PTV0.5 (green) and PTV0.7 (blue) were used to represent the three PMRM‐IMRT plans, respectively.

**Fig. 2:**
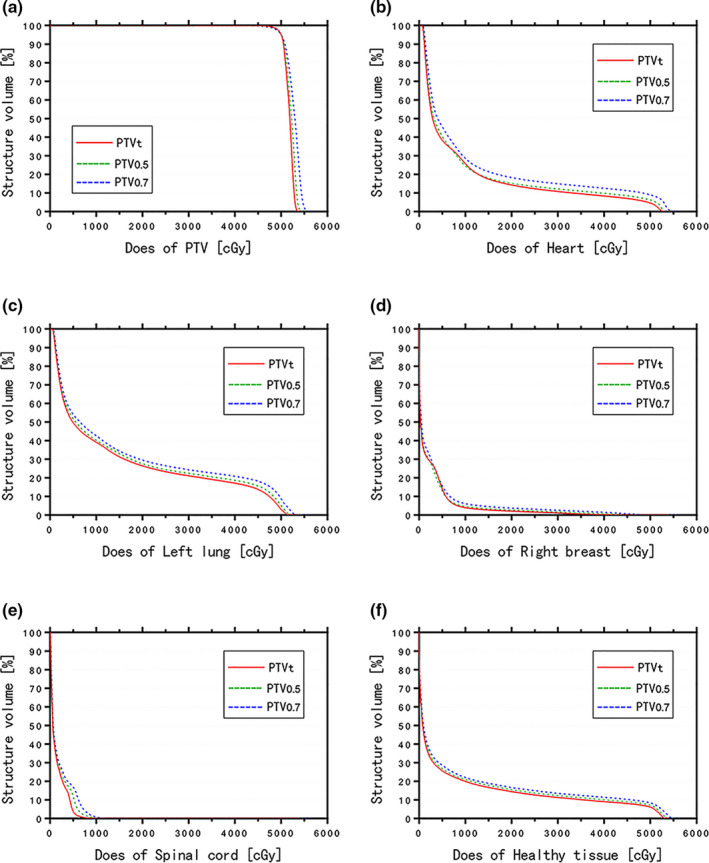
Comparison of dose volume histograms (DVHs) among the three PMRM‐IMRT plans using PTV_t_ (red), PTV_0.5_ (green), and PTV_0.7_ (blue), respectively. The charts showed the DVHs for PTV (a), heart (b), left lung (c), right breast (d), spinal cord (e) and healthy tissue (f).

We expanded CTV 3 mm in x, 4 mm in y, and 4 mm in z direction (the average value of the inter‐fractional CTV displacement without correction or optimization) to generate PTVt and created the corresponding IMRT plans. We have performed Shapiro‐Wilk test for each group of the raw data (including D_mean_, D_2%_, D_50%_, D_98%_, V_95%_, V_105%_, CI and HI of PTV, D_mean_, V_5_ _Gy_, V_10_ _Gy_, and V_20_ _Gy_ of Heart, D_mean_, V_5_ _Gy_, V_10_ _Gy_, and V_20_ _Gy_ of Left Lung, D_mean_ of Right Breast, D_max_ of Spinal Cord) and found that they are of the Gaussian distribution. The PMRM‐IMRT using PTV_t_ (V_95%_ = 99.57 ± 0.20, HI = 0.085 ± 0.01) provided perfect PTV dose coverage and the best homogeneity compared with those using PTV_0.5_ (V_95%_ = 99.30 ± 0.55, HI = 0.095 ± 0.01) or those using PTV_0.7_ (V_95%_ = 99.04 ± 0.29, HI = 0.117 ± 0.01). Moreover, the OARs including heart, left lung, right breast, spinal cord, healthy tissue in PMRM‐IMRT using PTV_t_ (heart‐D_mean_ = 9.01 ± 2.67 Gy, left lung‐D_mean_ = 14.47 ± 1.15 Gy, right breast‐D_mean_ = 3.09 ± 2.24 Gy, spinal cord −D_max_ = 5.76 ± 2.99 Gy, healthy tissue‐D_mean_ = 7.87 ± 1.19 Gy) had significantly lower radiation exposure compared with those using PTV_0.5_ (heart‐D_mean_ = 9.84 ± 2.67 Gy, left lung‐D_mean_ = 15.45 ± 0.95 Gy, right breast‐D_mean_ = 3.57 ± 2.46 Gy, spinal cord −D_max_ = 8.12 ± 3.48 Gy, healthy tissue‐D_mean_ = 8.48 ± 1.18 Gy) (all *P* < 0.01) or those using PTV_0.7_ (heart‐D_mean_ = 11.10 ± 2.97 Gy, left lung‐D_mean_ = 16.53 ± 1.22 Gy, right breast‐D_mean_ = 4.12 ± 2.85 Gy, spinal cord‐D_max_ = 9.76 ± 3.23 Gy, healthy tissue‐D_mean_ = 9.06 ± 1.19 Gy) (all *P* < 0.05). Additionally, PMRM‐IMRT plans using PTV_0.5_ also had satisfactory PTV dose coverage and better OARs including heart, left lung, right breast, spinal cord, healthy tissue sparing compared with those using PTV_0.7_ (all *P* < 0.01) (Figures 1, 2 and Table 3).

**Table 3:** Dosimetric comparison of PMRM‐IMRT for left‐sided breast cancer patients using PTV_t_ with those using PTV_0.5_ or those using PTV_0_._7_.

**Paramaters**	**PTV_t_**	**PTV_0.5_**	**PTV_0.7_**	**p1**	**p2**	**p3**
PTV						
D_mean_	51.83 ± 0.19	52.06 ± 0.24	52.43 ± 0.48	0.105	**0.018**	**0.004**
D_2%_	53.58 ± 0.35	54.05 ± 0.42	54.80 ± 0.52	**0.001**	**0.001**	**0.004**
D_50%_	51.94 ± 0.24	52.20 ± 0.33	52.66 ± 0.58	**0.006**	**0.003**	**0.002**
D_98%_	49.12 ± 0.18	49.07 ± 0.17	48.63 ± 0.33	**0.006**	**0.005**	0.274
V_95%_	99.57 ± 0.20	99.30 ± 0.55	99.04 ± 0.29	0.160	**0.001**	0.198
V_105%_	27.98 ± 9.45	39.47 ± 11.51	53.35 ± 17.41	**0.004**	**0.002**	**0.003**
HI	0.085 ± 0.01	0.095 ± 0.01	0.117 ± 0.01	**<0.001**	**<0.001**	**0.002**
CI	0.513 ± 0.05	0.550 ± 0.04	0.593 ± 0.04	**0.001**	**<0.001**	**0.001**
Heart						
D_mean_	9.01 ± 2.67	9.84 ± 2.67	11.10 ± 2.97	**<0.001**	**<0.001**	**<0.001**
V_5Gy_	36.78 ± 5.22	39.58 ± 5.15	43.17 ± 6.73	**0.044**	**0.009**	**<0.001**
V_10Gy_	25.30 ± 4.26	26.78 ± 4.47	29.76 ± 5.46	0.050	**0.023**	**0.046**
V_20Gy_	13.22 ± 2.53	14.48 ± 2.48	16.74 ± 3.57	0.056	**0.021**	**<0.001**
Left lung						
D_mean_	14.47 ± 1.15	15.45 ± 0.95	16.53 ± 1.22	**<0.001**	**<0.001**	**<0.001**
V_5Gy_	50.62 ± 1.76	52.78 ± 1.67	54.85 ± 3.26	0.079	**0.011**	**<0.001**
V_10Gy_	39.16 ± 1.95	41.18 ± 1.98	43.31 ± 2.89	**0.045**	**0.006**	**<0.001**
V_20Gy_	26.72 ± 1.12	28.66 ± 1.05	30.51 ± 2.44	0.067	**0.009**	**<0.001**
Right breast						
D_mean_	3.09 ± 2.24	3.57 ± 2.46	4.12 ± 2.85	**0.004**	**0.004**	**0.003**
Spinal cord						
D_max_	5.76 ± 2.99	8.12 ± 3.48	9.76 ± 3.23	**<0.001**	**<0.001**	**0.001**
Healthy tissue						
D_mean_	7.87 ± 1.19	8.48 ± 1.18	9.06 ± 1.19	**<0.001**	**<0.001**	**<0.001**

These data represent statistically significant data (p < 0.05), and bold is used to make it more obvious.

Abbreviations: PMRM‐IMRT = post modified radical mastectomy intensity‐modulated radiotherapy. PTV_t_ = tailored PTV expansion three‐dimensionally. PTV_0.5_ = PTV generated from isotropic 0.5‐cm expanding margin of CTV. PTV_0.7_ = PTV generated from isotropic 0.7‐cm expanding margin of CTV. Data presented as mean ± standard deviation. D_mean_ = mean dose (Gy). D_2%_ = the maximum dose. D_98%_ = the minimum dose. V_x_ = volume (%) receiving x dose (Gy) or higher. HI = heterogeneity index. CI = conformity index. P: p values from pair samples *t* test. p1: PTV_t_ & PTV_0.5_, p2: PTV_t_ & PTV_0.7_, p3: PTV_0.5_ & PTV_0.7_.

## 4 DISCUSSION

Increasing PTV expansion in post modified radical mastectomy intensity‐modulated radiotherapy (PMRM‐IMRT) for left‐sided breast cancer incredibly leads to the increasing radiation exposure of heart and left lung, which leads to the increasing risk of the long‐term radiation injury correspondingly.^2^, ^5^, ^8^, ^10^, ^11^ Considering setup and respiration motion uncertainties, either an isotropic 0.7‐cm or an 0.5‐cm expanding margin of CTV was referred to as an “approximate value” in published study^12^ and should not serve as the golden standard of PTV expansion for all patients. The CTV displacement could differ significantly among different individuals, among women in different treatment systems, among different phases of a respiratory cycle and among three different dimensional directions within the same patient. Such differences probably cannot be clinically neglected. Moreover, the inter‐fractional CTV displacement could be quite different in different treatment systems. More accurately quantifying and minimizing CTV displacement could potentially improve the dose distribution.

In our study, we measured the intra‐fractional CTV displacement^13^, ^14^ attributed to respiratory movement by defining nine points on the left chest wall and quantifying their displacement by using the 4D CT. We than measured the inter‐fractional CTV displacement resulting from the integrated effect of respiratory movement, thoracic disformation and setup errors by using the on‐board cone beam CT. Interestingly, we found that the CTV displacement in post modified radical mastectomy intensity‐modulated radiotherapy predominantly arises from inter‐fraction rather than from intra‐fraction during natural breathing, and differs in three directions either inter‐fractionally or intra‐fractionally. Tailoring and minimizing PTV expansion three‐dimensionally based on inter‐fractional CTV displacement significantly improves the dosimetry of PMRM‐IMRT for left‐sided breast cancer patients.

Clinical target volume displacement could be repeatedly quantified intra‐fractionally by 4D CT. Inter‐fractional CTV displacement could be repeatedly quantified by CBCT. The inter‐fractional CTV displacement attributed to the mixed effect of respiratory movement, thoracic disformation and setup errors, measured by on board cone beam CT was much larger than the displacement in the intra‐fraction and also differed in three dimensions under natural breathing, with 1.7 ± 1.2 mm in x direction, 2.8 ± 2.3 mm in y direction, and 2.6 ± 2.2 mm in z direction, respectively. Evidently, the PTV expansion of post modified radical mastectomy intensity‐modulated radiotherapy (PMRM‐IMRT) for left‐sided breast cancer patients should be based on the inter‐fractional CTV displacement rather than the intra‐fractional one during natural breathing. Therefore, we could not obtain respiration‐induced CTV displacement‐related dosimetric benefit when the real‐time respiratory gating technics, one of the most effective approaches to minimize radiation dose delivery to normal tissue and maximize delivery to tumors under patient's motion caused by respiration,^15^, ^16^, ^17^ was applied to PMRM‐IMRT for left‐sided breast cancer patients under natural breathing paradigm.

We expanded CTV 3 mm in x, 4 mm in y, and 4 mm in z direction (the average value of the inter‐fractional CTV displacement without correction or optimization) to generate PTV_t_ and created the corresponding IMRT plans. Our results indicate that PMRM‐IMRT plans for left‐sided breast cancer patients with PTV_t_ has dosimetrical advantages compared with plans with PTV_0.5_ and those with PTV_0.7_. given its perfect PTV coverage and better heart and left lung sparing. Additionally, the PMRM‐IMRT plans with PTV_0.5_ has dosimetrical advantages compared with plans with PTV_0.7_. owing to their better OARs sparing. Collectively, our data supported that tailoring and minimizing PTV expansion three‐dimensionally significantly improves the dosimetry of PMRM‐IMRT for left‐sided breast cancer patients.

Nevertheless, the average tailored PTV expansion without correction only covered 93.3% of all CTV in x, 63.3% in y, and 66.6% in z direction. To reach better CTV target coverage without increasing the expansion value when applying to clinical practice, we could do a successive of 3D on board cone beam CT verification and position correction inter‐fractionally at the first 2 weeks of radiotherapy for each patient, until the inter‐fractional CTV displacement was stably and repeatably less than the “tailored PTV expansion” value three‐dimensionally. Modality with more individualized tailored or minimized PTV expansion and the availability based on the displacement quantifying of the different parts of the chest wall, as well as the corresponding techniques applied in deep breathing^18^ warranted further study.

## 5 CONCLUSION

Our study exhibits that the CTV displacement in post modified radical mastectomy intensity‐modulated radiotherapy for left‐sided breast cancer patients predominantly results from inter‐fraction rather than from intra‐fraction during natural breathing, and differs in three dimensions either inter‐fractionally or intra‐fractionally. The respiratory gating technics was not recommended to be applied in PMRM‐IMRT for left‐sided breast cancer during natural breathing. Tailoring and minimizing PTV expansion three‐dimensionally based on inter‐fractional CTV displacement can significantly improve the dosimetry of PMRM‐IMRT for left‐sided breast cancer patients under this respiratory paradigm.

## ETHICAL DECLARATIONS

The study was performed in accordance with the Declaration of Helsinki, and was approved by the Ethics Committee of the Cancer Hospital of Shantou University Medical College. Informed consent form was obtained from each patient.

## AUTHOR CONTRIBUTIONS

Conception and design: Changchun Ma. Financial support: Changchun Ma, Jiyong Zhang. Provision of study materials or patients: Dongsheng Li, Fangcai Wu, Lili Wu, Guoxi Wang: Collection and assembly of data: Jiyong Zhang, Lei Huang, Baotian Huang. Data analysis and interpretation: Changchun Ma, Jiyong Zhang, Lei Huang, Yan Lin: Manuscript drafting: All authors. Final approval of manuscript: All authors.

## CONFLICT OF INTEREST

No conflict of interest.
